# Genome-Wide Identification and Functional Characterization of the Phosphate Transporter Gene Family in *Sorghum*

**DOI:** 10.3390/biom9110670

**Published:** 2019-10-30

**Authors:** Jiahui Wang, Yang Yang, Lingzi Liao, Jiawei Xu, Xiao Liang, Wen Liu

**Affiliations:** Key Laboratory of Three Gorges Regional Plant Genetics and Germplasm Enhancement (CTGU)/ Biotechnology Research Center, College of Biological and Pharmaceutical Sciences, China Three Gorges University, Yichang 443002, China

**Keywords:** *Sorghum*, phosphate transporters, gene family, gene expression, low phosphate

## Abstract

The phosphate transporter (PHT) family mediates the uptake and translocation of the essential macronutrient phosphorus (P) in plants. In this study, 27 PHT proteins in *Sorghum* were identified via bioinformatics tools. Phylogenetic analysis of their protein sequences in comparison with those family proteins from *Arabidopsis* and rice indicated that these proteins could be clustered into five typical subfamilies. There are 12 SbPHT1 members, one SbPHT2, six SbPHT3s, six SbPHT4s, and two SbPHOs in *Sorghum*. Further analysis of the gene structure, conserved motifs, subcellular localization, and transmembrane domains suggested that these features are relatively conserved within each subfamily. Meanwhile, the qRT-PCR assay implied that *SbPHT1;2*, *SbPHT1;11*, and *SbPHT4;6* were significantly upregulated in roots when exposed to low-phosphate conditions, suggesting that these genes might be involved in P uptake in low-phosphate conditions. Our study will increase our understanding of the roles of phosphate transporters in *Sorghum*.

## 1. Introduction

Phosphorus (P) is an essential nutrient for plant growth and development, contributing about 0.2% of the dry weight. It serves as an important component of many biological macromolecules, such as nucleic acids, membrane lipids, and ATP. Meanwhile, multiple plant biological processes, including photosynthesis, respiration, and glycolysis, require this element [1]. In plants, P is mainly taken up from the soil by the roots in the form of inorganic phosphate (Pi), including H_2_PO_4_^−^ and HPO_4_^2−^. Although P is abundant in the soil, the form of Pi, available for plant uptake, is relatively rare (0.1–10 μM). However, in the cytoplasm of plant cells, the Pi concentration is generally about 5–10 mM [2,3]. Thus, the uptake of P from the soil to the plant roots is an active process and requires special transporters.

Phosphorus uptake from soil and translocation between plant tissues and organs is facilitated by phosphate transporters (PHT), which are Pi:H^+^ symporters. The first PHT protein identified in higher plants was AtPT1 from *Arabidopsis thaliana* [4]. Since then, many genes encoding PHTs have been identified and characterized from a series of plant species, including *Arabidopsis thaliana* [5,6], *Oryza sativa* [7], *Triticum aestivum* [8], and *Solanum tuberosum* [9]. In addition, genome-wide identification of plant *PHT* family genes has also been reported in *Populus trichocarpa* [10,11], *Arabidopsis thaliana* [12,13], *Triticum aestivum* [14], *Solanum lycopersicum* [15], *Oryza sativa* [16], *Solanum tuberosum* [17], and *Malus domestica* [18].

There are two Pi uptake systems in plants: a low-Pi-inducible high-affinity system and a constitutive low-affinity system [3]. The former is inducible by and responsible for low Pi concentrations, whereas the latter is constitutively expressed. The plant genes encoding Pi transporters are classified into five families, *PHT1*, *PHT2*, *PHT3*, *PHT4*, and *PHO*, the first four of which are reported to localize in the plasma membrane, plastid inner membrane, mitochondrial inner membrane, and Golgi compartment, respectively [2,9]. Among the four families, the PHT1 family is relatively well characterized. Most PHT1 family members are high-affinity Pi transporters. Although the majority of *PHT1* genes are expressed in roots and responsible for Pi uptake from the soil under Pi-limited environments [5], many of them are also located in the leaves, stems, and flowers, suggesting that they may be involved in Pi translocation inside the plant [19,20]. In *Arabidopsis*, there are nine *PHT1* family genes (*AtPHT1;1-9*), some of which are regulated in response to changes in Pi availability [21]. In rice, 13 members of the *PHT1* family (*OsPT1-13*) contribute to Pi uptake from the soil and internal translocation [19,22]. In maize, *ZmPht1;1-5* are expressed both in roots and in shoots, and are induced by Pi starvation [23]. In soybean, the *PHT1* family is composed of 14 members (*GmPT1-14*), encoding high-affinity transporters and contributing to Pi uptake in roots under low-Pi conditions [24]. In *Medicago truncatula*, five *PHT1* genes are also induced by low Pi and are responsible for Pi uptake and translocation [25,26]. In contrast to the PHT1 family, the other families are less investigated and mainly contribute to Pi distribution within subcellular compartments [3]. The PHT2 family only has one member in many species, such as rice, *Arabidopsis*, and maize. AtPHT2;1 is a low-affinity transporter located in chloroplasts, responsible for phosphate-starvation responses in *Arabidopsis* [27]. In addition, there are 11 members of *PHO1* gene family in *Arabidopsis*, required for Pi translocation [6,12,28].

Aside from the PHT2 family, the other families are composed of more than one member. The Pi-affinity and expression pattern within the family vary greatly. For instance, *AtPHT1;6* is mainly expressed in floral organs, revealing the role of PHT1 transporters in flower development [5]; the other eight *AtPHT1s* are all at least expressed in the roots, in line with their major role in Pi uptake from the soil [29]. *AtPHT1;1* and *AtPHT1;4* are two high-affinity transporters and contribute to Pi uptake under both normal and low-Pi conditions [30]. In addition, *AtPHT1;5* is required for Pi translocation from roots to shoots [31]. The expression of *AtPHT1;8* and *AtPHT1;9* is inducible by low-Pi concentrations and functions in Pi translocation within plants [32,33]. Taken together, most PHT1 members in *Arabidopsis* are detected in diverse tissues or organs and share overlapping expression patterns with other PHT1 transporters, suggesting the functional redundancy and complexity of roles [5,29,34].

*Sorghum* (*Sorghum bicolor* (L.) Moench) is a C4 crop with high photosynthetic efficiency and environmental adaptability. As compared with its closely-related species maize, *Sorghum* is more resistant to harmful environments, including drought, high temperature, high salinity, and sterile soil. Although the whole-genome sequence of *Sorghum* has been published [34], the PHT family genes in *Sorghum* have not been systematically investigated yet. In this study, we identified 27 potential *SbPHT* family members via sequence alignment, classified these proteins into subfamilies, and further analyzed their sequences and expression patterns.

## 2. Materials and Methods

### 2.1. Plant Materials and Growth Conditions

The *Sorghum* genotype BTx623 was used in this study. For low-phosphate treatment, *Sorghum* seeds were first surface-sterilized with 70% ethanol for 2 min, followed by 5% NaClO for 20 min. Then, the seeds were washed with sterile water three times and planted individually on one-half-strength Murashige and Skoog (1/2 MS) medium with (control) or without K_2_PHO_4_ (low phosphate). Thereafter, the plants were placed in a greenhouse at 28 ± 2 °C under 12 h light of 100 μmol quanta m^−2^ s^−1^ irradiance and 12 h dark cycles.

### 2.2. Identification of Potential PHT Genes in Sorghum and Analysis of Their Chromosomal Localization

The potential *PHT* genes were isolated via homologous alignment. First, the *PHT* family genes from rice and *Arabidopsis* were obtained from the rice genome annotation project database (http://rice.plantbiology.msu.edu/) and the *Arabidopsis* Information Resource (TAIR) database (https://www.arabidopsis.org/), respectively. Then, the CDS query of each gene was blasted in the Phytozome database (https://phytozome.jgi.doe.gov/pz/portal.html) to search for *PHT* homologs in *Sorghum* with the *E*-value cutoff set as 1.00E−10. The top three matched genes were considered as candidates. Thereafter, we collected all the candidates and further checked their annotation information in the MOROKOSHI database (http://sorghum.riken.jp/morokoshi/Home.html). Finally, all the candidate protein sequences were further checked using NCBI’s conserved domain database (http://www.ncbi.nlm.nih.gov/cdd) and the Pfam protein family database (http://pfam.xfam.org). The *Sorghum PHT* genes were located on the corresponding chromosomes by the MapGene2chromosome web v2 (MG2C) database (http://mg2c.iask.in/mg2c_v2.0/) according to their position information, available on the Phytozome database (*Sorghum bicolor* v3.1.1).

### 2.3. Phylogenetic Analysis

The coding sequences (CDS) and protein sequences of *PHT* genes from rice, *Arabidopsis*, maize, and *Sorghum* were used for phylogenetic analysis. These sequences were downloaded from the rice genome annotation project database, the TAIR database, and the Phytozome database. Thereafter, multiple alignments of the CDS and protein sequences of *AtPHTs*, *OsPHTs*, and *SbPHTs* were carried out using ClustalX v1.83 software. Then, the unrooted phylogenetic trees were constructed using the neighbor-joining method with 1000 bootstrap iterations in MEGA5.05 software.

### 2.4. Gene Structure Analysis and Identification of Conserved Motifs

The mRNA sequences of the *Sorghum PHT* family genes were obtained from the Phytozome database (*Sorghum bicolor* v3.1.1). Then, the intron/exon distribution was determined by using the online gene structure display server program (GSDS, http://gsds.cbi.pku.edu.cn/). The conserved motifs in the full-length *Sorghum* PHT proteins were analyzed using multiple em (expectation maximization) for motif elicitation (MEME) version 5.0.3 (http://meme-suite.org/). The following parameters were applied: 20 motifs in maximum, 6–50 residues (inclusive) in motif width, and *E*-values <1.00E-40.

### 2.5. Protein Properties and Sequence Analysis

The isoelectric points (pIs) and molecular weights (MW) of candidate SbPHTs were detected using the ExPASy proteomics server database (http://expasy.org/). The transmembrane helices (TMHs) of the SbPHT proteins were predicted by using three online databases with their default settings, including the TMHMM Server v. 2.0 (http://www.cbs.dtu.dk/services/TMHMM/), the transporter classification database (http://www.tcdb.org/), and the TMbase (https://embnet.vital-it.ch/software/TMPRED_form.html).

### 2.6. Subcellular Localization

In this study, four online databases were used to predict the subcellular localization of each SbPHT protein, including CELLO v2.5 (http://cello.life.nctu.edu.tw/), Plant mPLoc (http://www.csbio.sjtu.edu.cn/bioinf/plant-multi/), WoLFPSORT (http://www.genscript.com/wolf-psort.html), and MultiLoc2 (http://abi.inf.uni-tuebingen.de/Services/MultiLoc2). Also, to obtain high confidence predictions, WoLFPSORT, MultiLoc2, and CELLO v 2.5 were set with cutoffs of confidence score >9, confidence score >0.7, and reliability score >4.0, respectively. Similar results were obtained from these databases.

### 2.7. Expression Analysis

For the expression profiles under low-phosphate conditions, the *Sorghum* seeds were surface-sterilized and then germinated on a control or low-phosphate 1/2 MS medium. After being grown for 14 days, the leaves and roots were collected separately and frozen immediately in liquid nitrogen. RNA isolation, cDNA synthesis, and qRT-PCR were performed as previously described [35,36,37]. For each experiment, at least three independent biological replicates and three technical repetitions were assayed. *PP2A* and *EIF4A* were chosen as internal controls. The relative expression levels of the corresponding genes in comparison to the housekeeping genes were used to draw the figure in Genesis software. The primers used for qRT-PCR are listed in Table S1.

## 3. Results

### 3.1. Genome-Wide Identification of PHT Family Genes in Sorghum

In this study, we identified a total of 27 phosphate transporter (PHT) family members in *Sorghum* via BLAST researches. The accession numbers, coding sequences, and encoded protein sequences of the predicted PHT family members were downloaded from the online Phytozome database; their annotation information and orthologs were obtained from the online MOROKOSHI database; their conserved domains were validated using the online CDD database and PFAM database. Based on the functional annotations, we confirmed that these proteins are potential transporter proteins (Table S2). Moreover, most *PHT* genes in *Sorghum* only have one transcript, except for Sb06g002800 (two), Sb02g026490 (three), Sb01g014790 (three), Sb02g032440 (12), and Sb03g011370 (two) (Table S2). For those genes with multiple alternative splicing, the main transcript was marked in red and underlined in Table S2 and used for further analysis.

### 3.2. Phylogenetic Analysis of PHT Family Genes from Sorghum, Arabidopsis, and Rice

**Figure 1:**
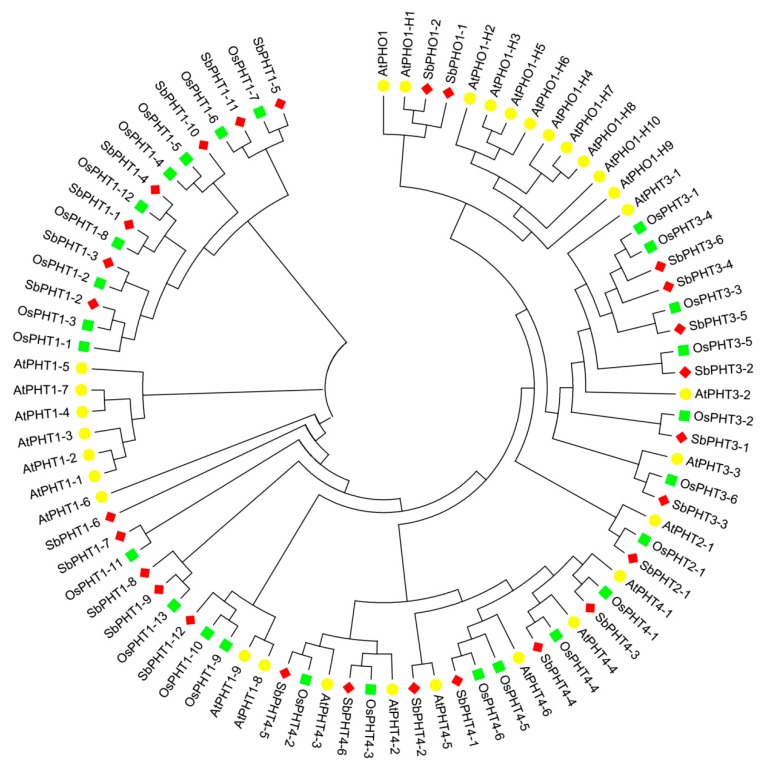
Phylogenetic tree of PHT proteins from *Sorghum*, *Arabidopsis*, and rice. The unrooted neighbor joining (NJ) phylogenetic tree was constructed by using the full-length sequences of PHT proteins from *Sorghum*, *Arabidopsis*, and rice. The red diamond, yellow circle, and green square represent PHT proteins from *Sorghum*, *Arabidopsis*, and rice, respectively.

Previous studies have revealed that the plant phosphate transporters include four PHT subfamilies (PHT1, PHT2, PHT3, and PHT4) and PHO [3,28]. To gain insight into the evolutionary relationships between PHT proteins as well as to classify *Sorghum* PHT proteins into subfamilies, we constructed an unrooted neighbor-joining (NJ) phylogenetic tree by comparing the protein sequences of known PHT proteins from *Arabidopsis* and rice. In total, we downloaded 26 OsPHT proteins, including 13 OsPHT1s, one OsPHT2, six OsPHT3s, and six OsPHT4s. Also, we obtained 30 AtPHT proteins, including nine AtPHT1s, one AtPHT2, three AtPHT3s, six AtPHT4s, and 11 AtPHTO1s. The locus names of PHT proteins from *Arabidopsis* and rice are listed in Table S3. Phylogenetic analysis (Figure 1) showed that 12 SbPHTs could be clustered into the SbPHT1 subfamily, which is the largest group in *Sorghum*. Meanwhile, the SbPHT3 and SbPHT4 subfamilies both have six members, the same as in rice. In addition, the SbPHT2 subfamily has only one member, similar to that in *Arabidopsis*, rice, and maize.

In general, *Sorghum* PHT proteins can be clustered into known subfamilies, indicating that this protein family may confer conserved functions in plants. On the other hand, the number and protein sequence of SbPHTs are more similar to OsPHTs than AtPHTs, suggesting that PHTs from *Sorghum* have a closer relationship with PHTs from rice as compared with those from *Arabidopsis*. Considering that *Sorghum* and rice are both dicotyledons, this observation is in accordance with the current understanding of plant evolutionary history. Based on phylogenetic analysis and their chromosome location, we have divided *SbPHT* genes into five subfamilies named *SbPHT1;1*–*SbPHT1;12*, *SbPHT2;1*, *SbPHT3;1*–*SbPHT3;6*, *SbPHT4;1*–*SbPHT4;6*, and *SbPHO1;1*–*SbPHO1;2*, respectively (Table 1).

### 3.3. Genomic Organization and Protein Properties of Sorghum PHT Proteins

**Figure 2:**
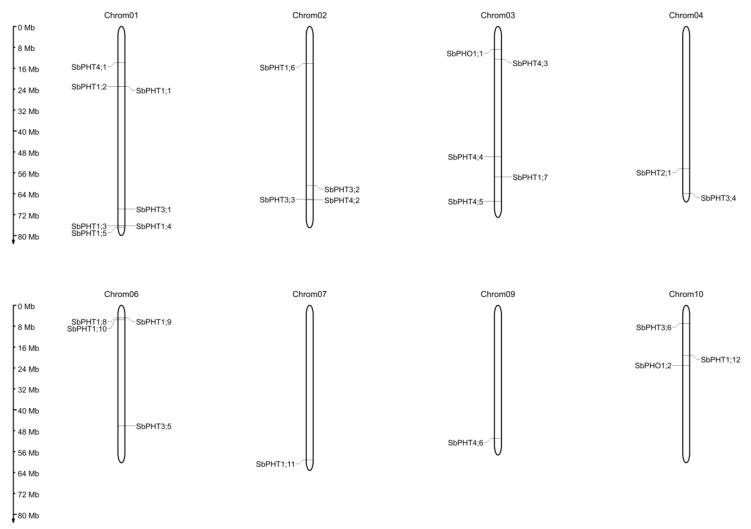
Distribution of *PHT* genes on the *Sorghum* chromosomes. The physical locations of the *Sorghum* PHT genes were obtained from the Phytozome database (*Sorghum bicolor* v3.1.1). Then, the location map was drawn with the MapGene2chromosome web v2 (MG2C) database (http://mg2c.iask.in/mg2c_v2.0/). The vertical bar on the left indicates the length of the *Sorghum* chromosomes in Mb. Chromosome numbers are indicated at the top of each column.

The physical locations of 27 *Sorghum* PHT proteins have been mapped on the *Sorghum* chromosomes according to their locus names obtained from the online Phytozome database. These 27 genes are located on eight chromosomes, excluding chromosomes 5 and 8 (Figure 2). To be exact, chromosome 1 contains the maximum seven *PHT* genes, followed by five on chromosome 2, four on chromosome 6, three on chromosome 10, two on chromosome 3, and a single gene each on chromosomes 7 and 9 (Figure 2). Generally, different subfamily genes are randomly distributed on the *Sorghum* chromosomes. Although most of the *SbPHT* genes are well spaced on the *Sorghum* chromosomes, there are also some potential tandem duplicates. For instance, Sb01g020570 (*SbPHT1;1*) and Sb01g020580 (*SbPHT1;2*), Sb01g046890 (*SbPHT1;3*) and Sb01g046900 (*SbPHT1;4*), and Sb06g002540 (*SbPHT1;8*) and Sb06g002560 (*SbPHT1;9*) are located in a series on chromosomes (Figure 2) and are highly homologous in terms of the protein sequence (Figure 1).

The length of presumed SbPHT proteins ranges from 318 (SbPHT3;3) to 852 (SbPHO1;2) amino acids (aa), with an average of 518.9 aa, and their relative molecular mass varies from 34.7 (SbPHT3;3) to 97.2 (SbPHO1;2) kDa (Table 1). Meanwhile, the theoretical isoelectric points (pIs) range from 5.85 (SbPHT4;2) to 9.92 (SbPHT4;5), with most of them around 9.0 and only four under 7.0 (Table 1). Generally, the protein size in each subfamily is quite close, with a difference less than 70 aa expected for the SbPHT4 subfamily. However, the theoretical pIs in each subfamily vary widely (Table 1).

### 3.4. Gene Structure and Conserved Motifs of Sorghum PHT Proteins

**Figure 3:**
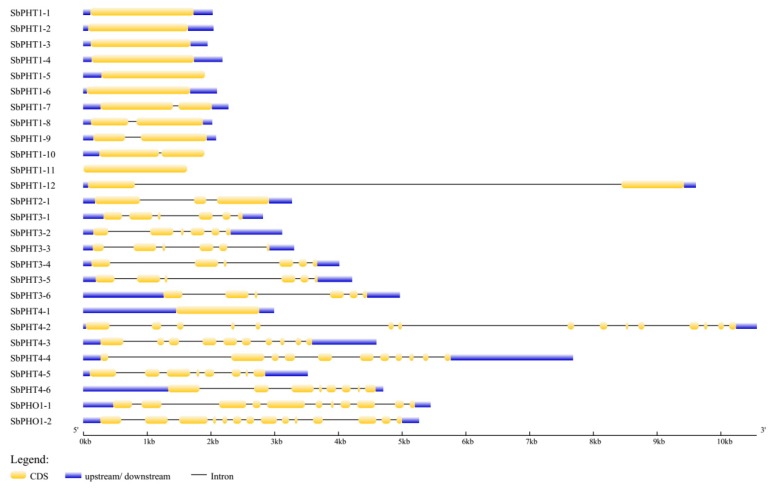
Gene structures of *PHT* genes in *Sorghum*. The full-length mRNA sequences of *SbPHT* genes are analyzed and displayed. The yellow boxes represent coding sequence (CDS), whereas the blue boxes represent upstream or downstream untranslated regions (UTRs). The introns in coding sequence are shown as black lines. The scale bar at the bottom indicates the length of the *SbPHT* genes in Kb.

**Figure 4:**
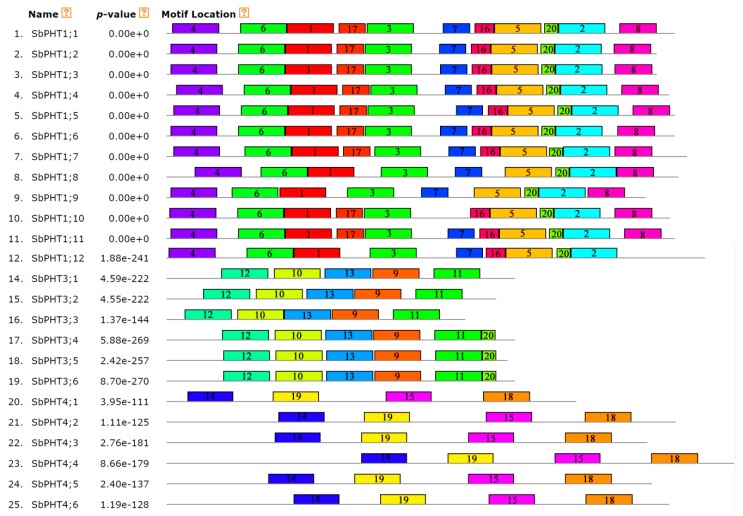
Analysis of the conserved motifs in the full-length *Sorghum* PHT proteins with the MEME program v5.0.3.

To gain insights into the structural features of predicted *SbPHT* genes, we determined the intron/exon distribution using an online gene structure display server program (GSDS, http://gsds.cbi.pku.edu.cn/). Our results showed that the *SbPHT1* subfamily genes have no more than one intron; *SbPHT2;1* has two introns, the *SbPHT3* subfamily genes have five introns, and the *SbPHO1* subfamily genes have more than 10 introns (Figure 3 and Table 1). However, the intron number in the *SbPHT4* subfamily genes varies greatly, from 0 to 14 (Figure 3). According to the intron/exon distribution data, we could demonstrate that the gene structures in each subfamily are relatively conserved, except for the *SbPHT4* subfamily. Then, we further analyzed the conserved motifs in the full-length *Sorghum* PHT proteins with the MEME program v5.0.3 (Figure 4). In total we identified 20 putative conserved motifs with 6–50 residues and *E*-values <1.00E-40. Among these 20 motifs, motifs 1–7 were present in all of the SbPHT1 subfamily proteins; motifs 9–13 only appeared in the SbPHT3 subfamily proteins; motifs 14, 15, 18, and 19 were in the SbPHT4 subfamily proteins; motifs 8, 16, and 17 were present in part of the SbPHT1 subfamily proteins; motif 20 was present in all the SbPHT1s and SbPHT3;4–SbPHT3;6. In general, the motifs are quite conserved within each subfamily.

### 3.5. Subcellular Localization and Transmembrane Domains of Sorghum PHT Proteins

As phosphate transporters, PHT proteins are reported to be membrane-bound [3]. Thus, we further predicted their transmembrane helices (TMHs) using the online databases. We found that SbPHT2;1 has the most TMHs, while the SbPHT3 subfamily members have no potential TMHs, as predicted by the TMHMM Server v. 2.0 (Table 1 and Table S4). Meanwhile, the subfamily members in SbPHT1 and SbPHO1 have 9–12 and 5–6 TMHs, respectively, whereas the TMH numbers in SbPHT4 vary from seven to 12 (Table 1). These results suggested that SbPHT proteins may also be membrane-bound, which is necessary for ion transport. Then, the subcellular localization of assumed *SbPHT* genes was predicted through four independent databases, Plant mPLoc, WoLFPSORT, MultiLoc2, and CELLO v2.5. To obtain high-confidence predictions, we set the cutoffs to confidence score >9, confidence score >0.7, and reliability score >4.0 for WoLFPSORT, MultiLoc2, and CELLO v 2.5, respectively. Our results indicated that most SbPHT1 and SbPHO1 subfamily members are likely located on the plasma membrane. However, there also have eleven proteins with confident but conflicting prediction results (Table S5), especially the SbPHT3 subfamily. Their subcellular localization needs to be investigated further.

### 3.6. Expression Profiles of Sorghum PHT Family Genes in Response to Low-Phosphate Conditions

**Figure 5:**
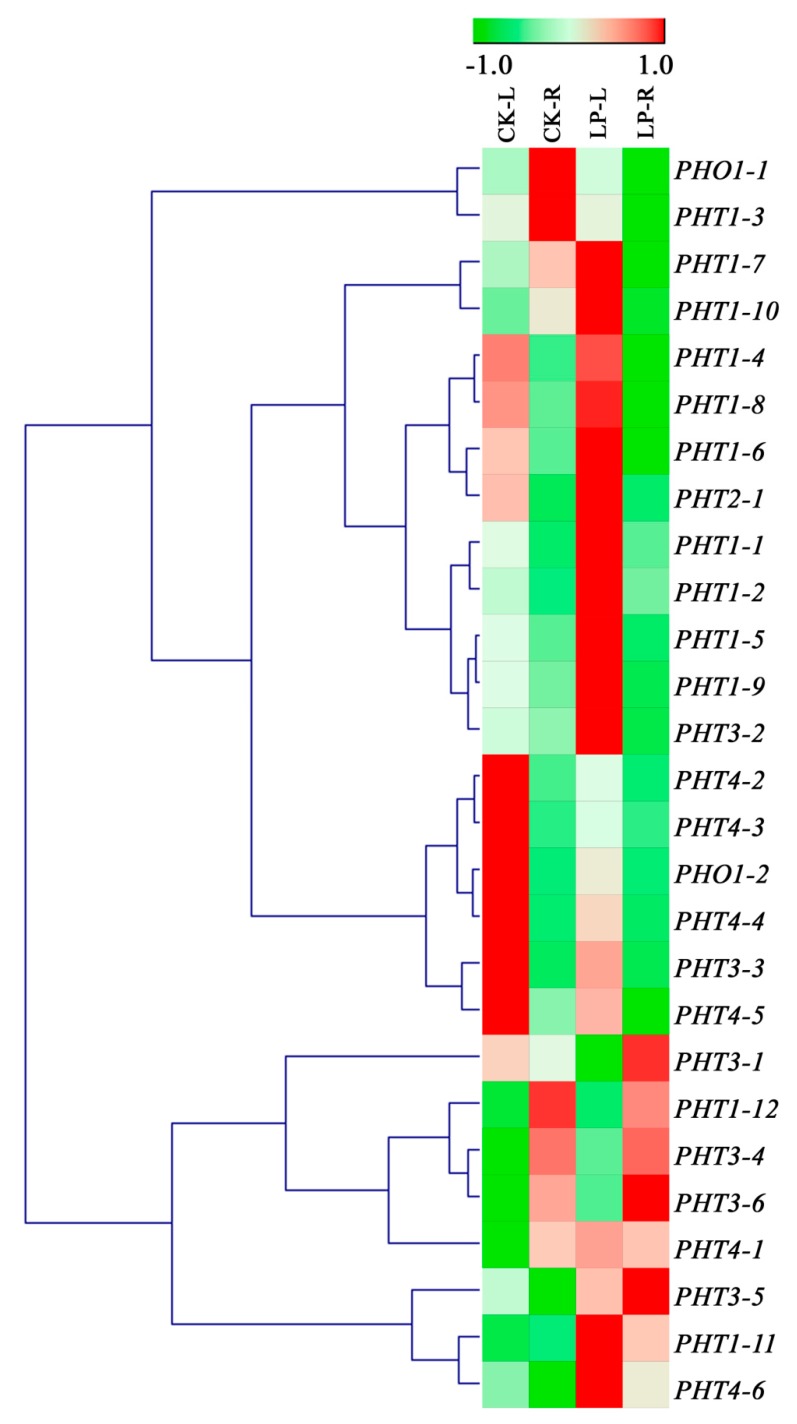
The relative expression levels of *SbPHT* genes in shoots and roots under normal and low-phosphate conditions. qRT-PCR analysis of *SbPHT* genes in the different tissues of 14-d-old *Sorghum* seedlings, including leaves under normal (CK-L) or low-phosphate (LP-L) growth conditions, and roots under normal (CK-R) or low-phosphate (LP-R) growth conditions. For each experiment, at least three independent biological replicates and three technical repetitions were assayed. *PP2A* and *EIF4A* were chosen as internal controls. The relative expression levels of the corresponding genes in comparison to the housekeeping genes were used to draw the figure in Genesis software. The red and green colors are presented by log transformation value with normalization.

The MOROKOSHI database, as well as the previously reported RNA-seq data [38,39,40,41,42], indicated the expression of SbPHTs in different tissues and under different growth conditions. However, their expression data in response to low-phosphate conditions are missing. To investigate the expression of *SbPHT* genes in response to phosphate starvation, we planted the *Sorghum* seeds on normal and low-phosphate 1/2 MS media for 14 days, and compared their relative expression levels under the above two growth conditions by qRT-PCR. Our data showed that 11 genes (*SbPHT1;1*, *SbPHT1;2*, *SbPHT1;5*, *SbPHT1;7*, *SbPHT1;9*, *SbPHT1;10*, *SbPHT1;11*, *SbPHT3;2*, *SbPHT3;6*, *SbPHT4;1*, *SbPHT4;6*) were upregulated more than 2-fold in the leaves after being phosphate-starved for 14 days (Figure 5). The other genes remained stable or were downregulated. In roots, only *SbPHT1;2*, *SbPHT1;11*, and *SbPHT4;6* were upregulated more than 2-fold upon low-phosphate treatment, while most genes remained stable (Figure 5). These results suggested that these upregulated genes might be involved in phosphate uptake when phosphate is limited in soils.

## 4. Discussion

*Sorghum bicolor*, which originated from Africa, is the fifth-largest crop in global cereal production, feeding over 750 million people, especially in arid and semi-arid areas [43]. As a C4 crop, it exhibits high photosynthetic efficiency, has a large biological and economic yield, and can be classified into four ideotypes, namely, grain, forage, energy, and sweet sorghum [44]. *Sorghum bicolor* is also highly tolerant to drought, flood, salinity and alkalinity, infertility, heat, and cold, and thus has been called “the camel of crops.” Currently, the full-length genome of *Sorghum bicolor* (inbred line BTx623) has been sequenced [34], which provides important gene resources for further functional genomics research and genetic improvement in crops. In this study, we isolated the phosphate transporters (PHT) gene family using bioinformatics approaches and analyzed their evolutionary and phylogenetic relationships, as well as expression patterns.

As key regulators of plant phosphate uptake, the PHT family has been previously reported in various plant species. For instance, Zhang and colleagues reported 42 *PHT* genes from the poplar, containing 14 *PtPHT1s*, two *PtPHT2s*, six *PtPHT3s*, eight *PtPHT4s*, and 12 *PtPHT5s* [11]. Sun et al. identified 37 *PHT* family members from the apple, including 14 *MdPHT1s*, two *MdPHT2s*, seven *MdPHT3s*, 11 *MdPHT4s*, and three *MdPHT5s* [18]. In the potato, 20 *StPHT* genes could be grouped into eight *StPHT1* homologs, one *StPHT2* homolog, two *StPHT3* homologs, five *StPHT4* homologs, and four *StPHO* homologs [17]. In addition, there are 26 and 30 *PHT* family genes in rice and *Arabidopsis*, respectively. In this study, we identified 27 putative *SbPHT* members based on sequence alignments with the *Arabidopsis* and rice *PHT* families (Figure 1 and Table S3). The distribution of the members in different subfamilies is quite similar in *Sorghum* and rice (Figure 1). According to current publications, the *PHT1* subfamily consisted of different members in different species, the number of which varied from four to 21 [14,16,17,18]. Although the total numbers of *PHT* family members differ between species, the *PHT1* and *PHT2* subfamilies are always the largest and smallest groups, respectively, implying that they may play a universal and a unique role in plants, respectively.

We noticed that Walder et al. previously reported 11 members belonging to *PHT1* in *Sorghum* [45]. Here, we found an additional *SbPHT1* member (*SbPHT1;12*, Sb10g012710), which exhibits high similarity to other *PHT1s*, not only in sequence similarity (Figure 1), but also in protein length, number of predicted transmembrane helices, and subcellular localization (Table 1). Analysis of the gene structures indicated that the numbers and distributions of exons and introns are relatively similar within each subfamily (Figure 3). These results further supported our clustering of the 27 SbPHT members into five subfamilies, mainly based on the phylogenetic tree (Figure 1).

As phosphate transporters, PHT proteins are reported to be membrane-bound [3,46]. Thus, we further analyzed the transmembrane domains of the identified 27 SbPHT proteins. To gain accurate results, we used three online databases with their default settings: the TMHMM Server v. 2.0 (http://www.cbs.dtu.dk/services/TMHMM/), the transporter classification database (http://www.tcdb.org/), and the TMbase (https://embnet.vital-it.ch/software/TMPRED_form.html). For most SbPHT members, the results from the above three databases are similar. However, the TMHMM server predicted that the SbPHT3 members had no TMHs, while the transporter classification database and TMbase predicted that they had 5–8 TMHs (Table S4). This might be due to those domains with a probability below 0.7 being excluded by the TMHMM server. The SbPHT3 members contained such low-probability TMHs. These predicted TMHs further supported the idea that these identified proteins are membrane-bound phosphate transporters in *Sorghum*.

The expression of *PHT* family members is inducible or constitutive [3]. Our qRT-PCR data indicated that multiply genes are induced in leaves under low-phosphate conditions, but most genes are stable in roots (Figure 5). To further reveal which gene(s) is responsible for phosphate uptake and translocation, we next need to examine the phosphate affinity of SbPHT proteins and construct mutants for gene functional analysis.

## 5. Conclusions

We have identified a total of 27 *PHT* family members in *Sorghum*; they could be clustered into five typical subfamilies. The protein features in each subfamily are very close, but their expression patterns are quite different.

## Figures and Tables

**Table 1:** Key features of *SbPHT* genes.

S. No.	Accession IDs	Gene IDs	Protein Length (aa)	Molecular Weight (kDa)	Isoelectric Point (pI)	Number of Introns	Number of Predicted Transmembrane Helices ^*^	Predicted Subcellular Location ^**^
*SbPHT1;1*	Sobic.001G234800	Sb01g020570	541	58.78	7.62	0	11	Plasma Membrane
*SbPHT1;2*	Sobic.001G234900	Sb01g020580	522	56.96	7.63	0	12	Plasma Membrane
*SbPHT1;3*	Sobic.001G502000	Sb01g046890	522	57.14	8.7	0	11	Plasma Membrane
*SbPHT1;4*	Sobic.001G502100	Sb01g046900	535	57.88	8.81	0	12	Plasma Membrane
*SbPHT1;5*	Sobic.001G513400	Sb01g047910	541	58.25	6.46	0	11	Plasma Membrane
*SbPHT1;6*	Sobic.002G116100	Sb02g009880	541	58.91	8.01	0	12	Plasma Membrane
*SbPHT1;7*	Sobic.003G243400	Sb03g029970	554	60.26	7.61	1	12	Plasma Membrane
*SbPHT1;8*	Sobic.006G026800	Sb06g002540	545	60.43	6.28	1	9	—
*SbPHT1;9*	Sobic.006G026900	Sb06g002560	510	56.26	9.31	1	12	Plasma Membrane
*SbPHT1;10*	Sobic.006G027300	Sb06g002800	536	58.63	6.8	1	10	Plasma Membrane
*SbPHT1;11*	Sobic.007G164400	Sb07g023780	541	57.80	8.88	0	11	—
*SbPHT1;12*	Sobic.010G133300	Sb10g012710	573	62.06	9.19	1	10	Plasma Membrane
*SbPHT2;1*	Sobic.004G199900	Sb04g024630	572	59.31	9.48	2	13	—
*SbPHT3;1*	Sobic.001G428500	Sb01g040260	371	39.73	8.97	5	0	—
*SbPHT3;2*	Sobic.002G224100	Sb02g026490	351	37.31	9.33	5	0	Chloroplast
*SbPHT3;3*	Sobic.002G291800	Sb02g032310	318	34.65	9.26	5	0	—
*SbPHT3;4*	Sobic.004G310300	Sb04g034260	371	38.970	9.3	5	0	—
*SbPHT3;5*	Sobic.006G097400	Sb06g018210	363	38.18	9.44	5	0	—
*SbPHT3;6*	Sobic.010G080900	Sb10g007010	371	39.13	9.22	5	0	—
*SbPHT4;1*	Sobic.001G169100	Sb01g014790	436	47.89	9.91	0	12	Plasma Membrane
*SbPHT4;2*	Sobic.002G293500	Sb02g032440	542	58.07	5.85	14	9	Plasma Membrane
*SbPHT4;3*	Sobic.003G133700	Sb03g011370	512	55.28	8.62	9	7	—
*SbPHT4;4*	Sobic.003G186800	Sb03g025190	604	66.41	9.73	10	11	Plasma Membrane
*SbPHT4;5*	Sobic.003G358900	Sb03g040080	517	55.66	9.92	7	10	—
*SbPHT4;6*	Sobic.009G157700	Sb09g022090	535	56.17	9.76	7	11	—
*SbPHO1;1*	Sobic.003G101000	Sb03g008460	851	95.45	9.04	10	5	Plasma Membrane
*SbPHO1;2*	Sobic.010G138800	Sb10g014220	852	97.17	8.78	13	6	—

*: predicted by the TMHMM Server v. 2.0 (http://www.cbs.dtu.dk/services/TMHMM/). **: — = undefined.

## Supplementary Materials

The following are available online at https://www.mdpi.com/2218-273X/9/11/670/s1, Table S1: The primers used for qRT-PCR, Table S2: IDs and annotations of *SbPHT* genes, Table S3: The locus names of PHT proteins from *Arabidopsis* and rice used for phylogenetic analysis, Table S4: Predication of the TMHs in SbPHT proteins via three online databases, Table S5: Subcellular predication of SbPHT proteins.
